# Isolation and HPLC Quantitative Determination of 5α-Reductase Inhibitors from *Tectona grandis* L.f. Leaf Extract

**DOI:** 10.3390/molecules27092893

**Published:** 2022-04-30

**Authors:** Kamonlak Insumrong, Kornkanok Ingkaninan, Neti Waranuch, Nutchaninad Tanuphol, Wudtichai Wisuitiprot, Trinop Promgool, Nungruthai Suphrom

**Affiliations:** 1 Department of Chemistry, Faculty of Science, Naresuan University, Phitsanulok 65000, Thailand; kamonlaki59@nu.ac.th; 2 Centre of Excellence in Cannabis Research, Department of Pharmaceutical Chemistry and Pharmacognosy, Faculty of Pharmaceutical Sciences and Center of Excellence for Innovation in Chemistry, Naresuan University, Phitsanulok 65000, Thailand; k_ingkaninan@yahoo.com (K.I.); nutchaninadt62@nu.ac.th (N.T.); 3 Cosmetics and Natural Products Research Center and Center of Excellence for Innovation in Chemistry, Department of Pharmaceutical Technology, Faculty of Pharmaceutical Sciences, Naresuan University, Phitsanulok 65000, Thailand; netiw@nu.ac.th (N.W.); tpromgool@gmail.com (T.P.); 4 Department of Thai Traditional Medicine, Sirindhorn College of Public Health, Phitsanulok 65130, Thailand; wisuitiprot@hotmail.com; 5 Department of Chemistry, Faculty of Science and Center of Excellence for Innovation in Chemistry, Naresuan University, Phitsanulok 65000, Thailand

**Keywords:** *Tectona grandis*, 5α-reductase inhibitor, diterpenes, quality control

## Abstract

Steroid 5α-reductase plays a crucial role in catalyzing the conversion of testosterone to dihydrotestosterone, which is involved in many androgen-dependent disorders. Leaf-hexane extract from *Tectona grandis* L.f. has shown promise as a 5α-reductase inhibitor. The objectives of this current study were to isolate and identify 5α-reductase inhibitors from *T. grandis* leaves and to use them as the bioactive markers for standardization of the extract. Three terpenoid compounds, (+)-eperua-8,13-dien-15-oic acid (**1**), (+)-eperua-7,13-dien-15-oic acid (**2**), and lupeol (**3**), were isolated and evaluated for 5α-reductase inhibitory activity. Compounds **1** and **2** exhibited potent 5α-reductase inhibitory activity, while **3** showed weak inhibitory activity. An HPLC method for the quantitative determination of the two potent inhibitors (**1** and **2**), applicable for quality control of *T. grandis* leaf extracts, was also developed. The ethanolic extract showed a significantly higher content of **1** and **2** than found in the hexane extract, suggesting that ethanol is a preferable extraction solvent. This study is the first reported isolation of 5α-reductase inhibitors (**1** and **2**) from *T. grandis* leaves. The extraction and quality control methods that are safe and useful for further development of *T. grandis* leaf extract as an active ingredient for hair loss treatment products are also reported.

## 1. Introduction

Testosterone is the major circulating androgen in many androgen-sensitive tissues that circulate androgen in the serum of men [1] and is ultimately synthesized by the Leydig cells of the testes under the control of the hypothalamus and anterior pituitary gland [2]. Testosterone can be metabolized in most tissues to dihydrotestosterone (DHT) by the enzyme steroid 5α-reductase where nicotinamide adenine dinucleotide phosphate (NADPH) is used as the cofactor [3]. The isoforms of human steroid 5α-reductase are distributed into three major isoforms (type 1–3) which are specifically located in human tissues [3,4]. The overproduction of DHT can cause several androgen-dependent disorders including androgenic alopecia (AGA), benign prostatic hyperplasia (BPH), prostate cancer, female hirsutism, and acne [5,6]. 

Androgenic alopecia (AGA), or male pattern baldness, is the most common form of hair loss affecting large numbers of both men and women [7,8]. This type of hair loss is heritable; androgen-dependent, which is associated with 5α-reductase type 1; and occurs in a defined pattern. Thus, the anti-androgens which exhibit inhibitory activity on 5α-reductase and/or block androgen receptors may be useful for the treatment of AGA. To date, only two drugs, finasteride (5α-reductase type 2 inhibitor) and minoxidil (vasodilator), have been approved by US Food and Drug Administration (FDA) for treatment of AGA [9]. However, finasteride can only be used orally and might cause some adverse effects such as impotence, abnormal ejaculation, decreased ejaculatory volume, abnormal sexual function, gynecomastia, testicular pain, and impairment of muscle growth [10,11], while topical minoxidil may cause irritation, itching, and allergic contact dermatitis on the scalp [12]. Given the seriousness and widespread nature of the problem, a search for natural ingredients that have 5α-reductase inhibitory activity is an important consideration.

*Tectona grandis* L.f. (teak) belongs to the family Lamiaceae and is locally known as the teak tree (English) or sak (Thai). This plant is a large deciduous tree reaching over 30 m and distributed in south and southeast Asia, mainly in India, Indonesia, Laos, Myanmar, and northern Thailand. Teak has a worldwide reputation as a quality timber on account of its remarkable physical and mechanical properties, particularly elasticity, strength, durability, and decay resistance [13,14]. Besides its economic importance source, *T. grandis* also plays a crucial role in traditional systems of medicine in India. Several parts of *T. grandis* are traditionally used for anti-inflammation, promoting hair growth, treatment of skin diseases, bronchitis, and retention of urine [15,16]. Some classes of the compound, including quinones (naphthoquinones and anthraquinones) [17,18,19], terpenoids [20], apocarotenoids [21], phenolic compounds [22], steroids or saponins [20,23], phenylpropanoids (lignans and norlignans) [24], phenylethanoid glycosides [25], and fatty esters [26], have been reported as chemical constituents in some parts of *T. grandis* (i.e., seeds, barks, woods, leaves, roots, and fruits). The *T. grandis* seeds are traditionally recommended as a hair tonic in the Indian system of medicine. The effect of *T. grandis* seeds on hair growth has been scientifically proven in the albino mice model. Treatment with 5% and 10% petroleum ether extracts has a greater ability to increase the number of hair follicles than the standard positive drug minoxidil [27].

Recently, our research group have investigated the biological activities of *T. grandis* extracts related to hair loss treatment. Among the extracts from several parts of *T. grandis*, leaf hexane and ethyl acetate extracts displayed potent 5α-reductase inhibition with the leaf hexane extract showing lower cytotoxicity on HFDPCs than that of ethyl acetate extract. Hexane extract (25 µg/mL) showed an anti-testosterone activity profile similar to finasteride, the positive control, and also demonstrated the inhibition of IL-1*β* secretion in lipopolysaccharide (LPS)-stimulated RAW 264.7 cells [28]. This discovery suggests that the leaf extract of *T. grandis* might serve as ingredients in alternative medicines or cosmetics for hair loss treatment. The objectives of the present study were therefore to isolate and identify the 5α-reductase inhibitors from *T. grandis* leaf-hexane extract. The 5α-reductase inhibitors isolated were to be used as markers for quality control of *T. grandis* leaf extracts.

## 2. Results and Discussion

### 2.1. Extraction and Isolation of Bioactive Compounds

**Figure 1:**
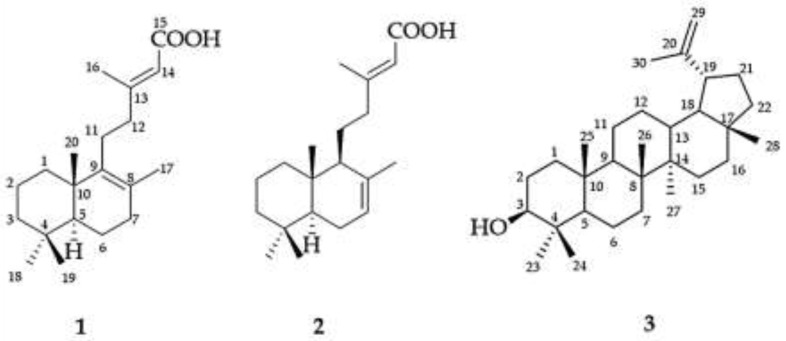
Chemical structures of three compounds isolated from *T. grandis* leaf extract.

The *T. grandis* leaf-hexane extract was first fractionated by the addition of chilled acetone to remove gum components. The gum component-free part (acetone soluble portion) was further dried and re-dissolved with MeOH to yield a crude extract (hTG). To isolate the bioactive constituents from the hTG extract, an in vitro 5α-reductase assay-guided fractionation of various fractions was initially carried out. Chromatographic fractionation and purification of the hTG extract yielded three known compounds. The structures of all isolated compounds were elucidated based on spectroscopic data, as shown in supplementary data (Figures S1–S14, Tables S1 and S2, See Supplementary Materials), and their spectra were also compared with the reported data [29,30,31,32]. Their structures (Figure 1) were thus identified as two eperuane-type diterpenes, namely (+)-eperua-8,13-dien-15-oic acid (**1**) and (+)-eperua-7,13-dien-15-oic acid (**2**), as well as a lupane-type triterpene, lupeol (**3**).

Various classes of terpenoids in *T. grandis* leaves and bark have been previously reported, such as sesquiterpenes (eudesmane- and oppositane-types), diterpenes (phytane- and eperuane-types), and triterpenes (ursane-, oleanane-, and lupane-types) [20,33].

According to the literature, compounds **1** and **2** are naturally occurring and chemically prepared compounds. They were first isolated and identified as the chemical constituents in the MeOH extract of *Sindora siamensis* Miq. leaves [29]. Moreover, **1** has also been synthesized from sclareol by superacidic cyclization of alcohols [34,35,36] whereas **2** could be synthesized by MnO_2_ and AgNO_3_ oxidation of labda-7,13 *E*-diene-15-ol [37], while (-)-eperua-7,13-dien-15-oic acid, the enantiomer of **2**, had been isolated from *Hymenaea coubarril* [31], *Isodon scoparius* [38], and *Copaifera langsdorffii* [39]. In addition, compound **3** was previously isolated from various plants such as *T. grandis* [33], *Wrightia tinctoria* R.Br. [40], *Oxystelma esculentum* (L. f.) Sm. [41], and *Taraxacum officinale* (L.) Weber ex F.H.Wigg. [42]. It should be noted that the present study described the isolation and identification of two eperuane-type diterpenes (**1** and **2**) as the chemical constituents in *T. grandis* for the first time.

### 2.2. Steroid 5α-Reductase Inhibitory Activity

The steroid 5α-reductase inhibitory activity of samples on the conversion of testosterone to DHT was determined using LNCaP cell as a source of enzyme. In the screening, the percentage of enzymatic inhibition of *T. grandis* leaf extracts and three isolated compounds were measured at the final concentration of 100 µg/mL. All samples showed a steroid 5α-reductase inhibitory effect greater than 80% inhibitory activity. Therefore, the concentrations that could inhibit 50% of enzymatic activity (IC_50_) of all samples were further determined. Two reference 5α-reductase inhibitors, finasteride and curcumin, were determined using our assay system as described in Srivilai et al. (2017) [43]. Their IC_50_ values on 5α-reductase are summarized in Table 1.

The results showed that inhibition of 5α-reductase by ethanolic extract (IC_50_ = 23.91 ± 0.17 µg/mL) was more potent than hexane extract (IC_50_ = 26.45 ± 0.69 µg/mL). Two isolated diterpenes (**1** and **2**) had the potent ability to inhibit 5α-reductase at IC_50_ value of 1 of 14.19 ± 2.87 µM (or 4.31 ± 0.87 µg/mL) and, for **2**, 14.65 ± 0.31 µM (or 4.45 ± 0.10 µg/mL). The 5α-reductase inhibitory activity of both **1** and **2** was significantly higher than a triterpene (**3**) but less than a standard 5α-reductase inhibitor, finasteride. Interestingly, there was no significant difference in the inhibitory activity between two potent compounds (**1** and **2**) and a positive control, curcumin. The limited number of compounds restricted the interpretation of structure–activity relationships. However, the presence of α,β unsaturated carboxylic acid in the side chain of **1** and **2** might be important for 5α-reductase inhibitory activity, while the less inhibitory activity of **3** is still largely unknown. Other factors might have been involved in the activity, which need to be investigated further. The importance of the carboxyl group for 5α-reductase inhibition has also been mentioned in the literature [44,45]. For example, ganoderic acid TR showed stronger inhibitory activity than 5α-lanosta-7,9(11),24-triene-15α,26-dihydroxy-3-one. The only difference in these two compounds is the position of C-26 which is a carboxyl group for ganoderic acid TR, and the hydroxyl group for 5α-lanosta-7,9(11),24-triene-15α,26-dihydroxy-3-one. These results demonstrated that a carboxyl group of 17*β*-side chain of ganoderic acid TR is important to elicit the inhibitory activity. Meanwhile, the methyl ester of ganoderic acid TR showed much less inhibitory activity on 5α-reductase. Additionally, the presence of unsaturated at C-24 and C-25 of three most potent inhibitors (ganoderic acid TR, ganoderic acid DM, and 5α-lanosta-7,9(11),24-triene-15α,26-dihydroxy-3-one) was imperative to their activity, while the fully saturated triterpenoids was less potent. The study of Srivilai et al. [46] also discussed the crucial role of α,β unsaturated ketone in sesquiterpenes for 5α-reductase inhibition.

For the pharmacological properties of these isolated compounds, **1** and **2** have been shown to possess histone deacetylase (HDAC) inhibitory activity [29], while **3** has shown anti-inflammatory activity [47], antimalarial activity [48], andapoptogenic activity [49,50], and to exhibit strong androgen receptor inhibitory activity [51]. Although the anti-androgenic effect via the androgen receptor inhibition by **3** had been reported, the 5α-reductase inhibitory activity of **1** and **2** had never been described before. The 5α-reductase activity of these compounds is reported here for the first time.

For the further development of products containing *T. grandis* extract for medical or cosmetic applications, standardization and quality control are necessary to guarantee consistent levels of bioactive compounds in the extract. We then carried out a quantitative study of the two bioactive markers (**1** and **2**) in the extracts prepared from hexane, as the previous report suggested, and ethanol, which was more economic and environmentally friendly.

### 2.3. Quantitative HPLC Analysis of 5α-Reductase Inhibitors from T. grandis Leaf Extract

The HPLC method for the quantitative determination of the two active compounds **1** and **2** was developed and validated according to ICH guidelines. The wavelength for quantitative determination at 220 nm was chosen to obtain the baseline separation of **1** and **2** when used as markers.

**Figure 2:**
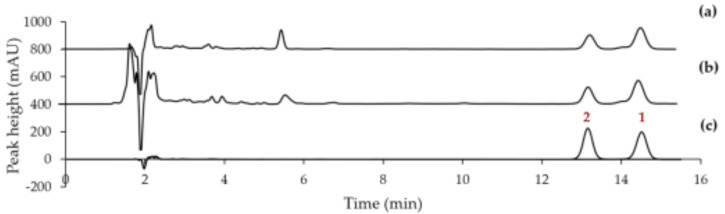
HPLC-DAD chromatograms of (**a**) 100 µg/mL *T. grandis* leaf-hexane extract, (**b**) 100 µg/mL *T. grandis* leaf-ethanolic extract, and (**c**) a mixture of 50 µg/mL isolated compounds **1** and **2**.

An isocratic elution of acetonitrile–formic acid in purified water as the mobile phase was conducted to successfully separate compounds **1** (t*_R_* 14.52 min) and **2** (t*_R_* 13.15 min) in the hexane and ethanolic extracts of *T. grandis* leaf within 15 min (Figure 2). The results of the method validation parameters for the determination of **1** and **2** are summarized in Table 2.

As a result, the plot of peak area versus the concentrations (1.56–200 µg/mL) of **1** and **2** provided good linearity for this method, with r^2^ of 0.9997 for **1** and 0.9995 for **2**. The LOD of **1** was 0.09 µg/mL and **2** was 0.06 µg/mL, while the LOQ of **1** was 0.30 µg/mL and **2** was 0.20 µg/mL, indicating a high sensitivity of the method.

The analytical method developed for the quantification of **1** and **2** had good accuracy, with the overall recovery in the range of 92.78–100.6%. The RSD values were less than 3% for the intra-day and inter-day which demonstrated the high precision of the method (Table 3). These results showed that the developed quantitative method was sensitive, accurate, and precise to determine two active constituents in the *T. grandis* leaf-extracts simultaneously.

The contents of the two 5α-reductase inhibitors, **1** and **2**, in hexane and ethanolic extracts of *T. grandis* leaf, were investigated using our validated HPLC method, previously discussed (see Table 2 and Table 3 for details). The peak identification of these components was characterized by comparison with the retention time of the reference compounds (Figure 2c). The results revealed that the ethanolic extract exhibited significantly higher contents of **1** (6.18 ± 0.12 % (*w*/*w*) and **2** (3.83 ± 0.04 % (*w*/*w*), while the hexane extract contained 5.60 ± 0.05 % (*w*/*w*) of **1** and 3.23 ± 0.03 % (*w*/*w*) of **2** (Table 4). This is in agreement with the fact that higher 5α-reductase inhibitory activity was found in the ethanolic extract. In addition, the ethanolic extract contained a lower amount of undesirable gum compared to the hexane extract (data not shown).

Moreover, ethanol is safe and has wide acceptability as an extraction solvent and ingredient in food, drugs, and cosmetics [52,53,54]. Therefore, 95% ethanol is recommended as a solvent for extracting *T. grandis* leaves with the further application as an ingredient in the drugs and cosmeceutical products for hair-loss treatment.

## 3. Materials and Methods

### 3.1. General Experimental Procedures

Thin layer chromatography (TLC) analysis was performed on TLC silica gel 60 F254 aluminum sheet 20 × 20 cm (Merck, Darmstadt, Germany). A silica gel column (0.040–0.063 mm granule size), a sephadex LH-20 column (particle size dry 18–111 µm), and a C18 column (40–63 particle size) were used for chromatographic isolation of the extract constituents. Fourier-transform infrared (FT-IR) spectra were recorded with attenuated total reflectance (ATR) mode on a PerkinElmer spectrum GX (Perkin Elmer, Waltham, MA, USA). Optical rotations were measured using a POLAX-2L polarimeter (Atago, Japan). An Agilent 1260 infinity high performance liquid chromatography instrument via an electrospray ionization (ESI) interface to a 6540 ultrahigh definition accurate mass Q-TOF (Agilent Technologies, Palo Alto, CA, USA) was conducted. Nuclear magnetic resonance (NMR) spectra were recorded on a Bruker AV400 (Bruker, Billerica, MA, USA) spectrometer at 400 MHz for proton and 100 MHz for carbon. The absorbance was measured using hybrid Multi-Mode Detection Synergy H1 (Model H1MF) (Bio-TeK Instruments, Winooski, VT, USA). The high performance liquid chromatography (HPLC) was performed using Agilent Technology (model 1260 infinity with fraction collector, Santa Clara, CA, USA).

### 3.2. Plant Material

Fresh mature leaves of *T. grandis* were collected from Banna district, Nakhon Nayok Province, Thailand in September 2019. The plant material was identified by Assist. Prof. Dr. Pranee Nangngam, Faculty of Science, Naresuan University. The voucher specimen (collection no. 05721) was deposited at the Department of Biology, Faculty of Science, Naresuan University, Phitsanulok, Thailand.

### 3.3. Extraction and Isolation

The fresh mature leaves of *T. grandis* (TG) were chopped into small pieces and dried at 55 °C. The dried material was ground into a fine powder and passed through a 60-mesh sieve. The *T. grandis* leaf powder was extracted individually using two organic solvents; hexane and 95% ethanol. For the preparation of crude hexane extract, the *T. grandis* leaf powder (1.5 kg) was macerated with hexane (6.0 L) three times (at least five days each time) at room temperature with occasional shaking, and the solvent was removed under reduced pressure to produce a dark green viscous crude hexane extract (127 g, 8.47% yield). For the preparation of crude ethanolic extract, a 292 g sample of the leaf powder was macerated with 95% ethanol (1.17 L) and, following the same procedure previously described, a dark green viscous crude ethanolic extract (32.90 g, 11.27% yield) was produced. Both of the resultant crude extracts were stored at −20 °C until used.

To isolate the 5α-reductase inhibitors, a 100 g sample of the hexane extract was dissolved in hexane (100 mL) to give hexane solution. The solution was dropped into 100 mL of chilled acetone to provide a 47.00 g acetone-soluble portion and a 54.56 g acetone-insoluble residue. The 47.00 g acetone-soluble portion was then dried and re-dissolved in methanol (MeOH) to give a methanol-soluble portion (hTG, 33.08 g) and methanol-insoluble residue (13.01 g). An 18.46 g sample of hTG was further fractionated on a sephadex LH-20 column (2.5 × 73 cm) using MeOH as the mobile phase to yield seven fractions (hTG.A–hTG.G). To identify the active constituents, activity-guided fractionation using an in vitro 5α-reductase inhibitory activity assay of the obtained fractions was performed. The most potent 5α-reductase inhibitory activity was detected in the hTG.B, part of which (9.85 g) was further chromatographed over a 2 × 88 cm sephadex LH-20 column using MeOH as eluent to provide six fractions (hTG.B1–hTG.B6). These fractions were also tested for 5α-reductase inhibitory activity. The most potent inhibitory activity was observed in two major fractions, hTG.B3 and hTG.B4. One of the active fractions, hTG.B4 (orange viscous, 5.06 g), was re-chromatographed over a silica gel column (5 × 13 cm) and eluted with increasing proportions of hexane and ethyl acetate (EtOAc) (99.5:0.5 to 0:100% *v*/*v*) to give seven fractions (hTG.B4/1-hTG.B4/7). Sub-fraction hTG.B4/3 (529.9 mg) was further isolated on a silica gel column (1.5 × 13 cm) and eluted with gradient ratio of hexane and EtOAc (95:0.5 to 0:100% *v*/*v*) to yield seven fractions (hTG.B4/3.1-hTG.B4/3.7). A white solid of hTG.B4/3.1 (90.0 mg) was then subjected to a reversed-phase C18 column (1.5 × 20 cm) with MeOH, giving three fractions (hTG.B4/3.1.1-hTG.B4/3.1.3). A white solid powder of hTG.B4/3.1.1 (61.1 mg) was re-chromatographed by HPLC (Agilent 1260 infinity with fraction collector, USA) on a Phenomenex Luna^®^ C18(2) column (250 × 10 mm, 10 µm particle size) under the following conditions: injection volume, 50 µL; flow rate, 5 mL/min; detection, 220 nm; mobile phase, acetonitrile:water (90:10 *v*/*v*). Two pure compounds; **1** (19.5 mg, *t*_R_: 11.03 min) and **2** (16.3 mg, *t*_R_: 10.12 min) were obtained. As well, an active fraction hTG.B3 (brownish syrupy, 1.71 g) was also fractionated using a sephadex LH-20 column (2 × 88 cm, MeOH) to provide four fractions (hTG.B3/1-hTG.B3/4). Fraction hTG.B3/3 (319.3 mg) was then loaded using silica gel column chromatography and sequentially eluted with a gradient formed of hexane, dichloromethane (CH_2_Cl_2_), and MeOH to give nine fractions (hTG.B3/3.1–hTG.B3/3.9). Fraction hTG.B3/3.3 (6.1 mg) was purified over a reversed-phase C18 column (1.5 × 20 cm, MeOH) to give colorless needle-shaped crystals, which yielded **3** (3.7 mg). Finally, compound **3** (1.7 mg) was also obtained from the fractionation of hTG.B3/3.4 (5.4 mg) using the same procedure for hTG.B3/3.3. The structures of all isolated compounds were elucidated by spectroscopic analysis and by comparing them with those reported in the literature.

(+)-Eperua-8,13-dien-15-oic acid (**1**): white solid; C_20_H_32_O_2_; mp 121–122 °C; ${\left[\alpha \right]}_{D}^{25}$ +80 (*c* 1.25, MeOH); UV (MeOH) λ_max_ (log ε) 209 nm (6.71); FT-IR (ATR) ν_max_ 2919, 2844, 1688, 1634, 1440 cm^−1^; HRESI-MS: *m*/*z* 303.2374 [M-H]^¯^ (Calcd for C_20_H_31_O_2_, 303.2403); ^1^H NMR (CDCl_3_, 400 MHz), ^13^C NMR (100 MHz, CDCl_3_) data (see Figures S1 and S2 and Table S1, Supplementary Data).

(+)-Eperua-7,13-dien-15-oic acid (**2**): colorless solid; C_20_H_32_O_2_; mp 101–102 °C; ${\left[\alpha \right]}_{D}^{25}$ +80 (*c* 1.25, MeOH); UV (MeOH) λ_max_ (log ε) 216 nm (6.72); FT-IR (ATR) ν_max_ 2959, 2922, 2841, 1693, 1634, 1455 cm^−1^; HRESI-MS: *m*/*z* 303.2352 [M-H]^¯^ (Calcd for C_20_H_31_O_2_, 303.2403); ^1^H NMR (CDCl_3_, 400 MHz), and ^13^C NMR (100 MHz, CDCl_3_) data (see Figures S6 and S7 and Table S1, Supplementary Data).

Lupeol (**3**): needle-shaped crystals; C_30_H_50_O; mp 212–215 °C; ${\left[\alpha \right]}_{D}^{25}$ +43 (*c* 0.23, CHCl_3_); UV (CHCl_3_) λ_max_ (log ε) 283 nm (4.81); FT-IR (ATR) ν_max_ 3347, 3066, 2941, 2871, 2852, 1637, 1451, 1378, 1188, 1105, 1035, 878, 688, 639, 545 cm^−1^; EI-MS *m*/*z* 426.39 [M]^+^; ^1^H NMR (CDCl_3_, 400 MHz), and ^13^C NMR (100 MHz, CDCl_3_) data (see Figures S11 and S12 and Table S2, Supplementary Data). The identification of **3** (purity 97%) was also performed by computer matching its recorded mass spectrum with the Wiley7n standard library and with data found in the literature.

### 3.4. Measurement of 5α-Reductase Inhibitory Activity

#### 3.4.1. Enzyme Preparation

5α-reductase was prepared as a crude enzyme from the androgen-dependent LNCaP cells (ATCC^®^ CRL-1740^TM^) using the procedure as described by Fachrunniza et al. [28], which was modified from Srivilai et al. [55]. Briefly, the LNCaP cells were cultured in a 175 cm^2^ culture flask at 37 °C under a 5% CO_2_ humidified atmosphere and cultured until reaching approximately 80% confluency. The medium was removed and the cells were rinsed with tris-HCl buffer solution, pH 7.4 (containing 10 mM Tris-HCl buffer; 50 mM KCl; 1 mM EDTA; 0.5 mM phenylmethanesulfonyl fluoride). The cells were then scraped off and centrifuged at 1900× *g* for 10 min. The cell pellets were collected and re-suspended in tris-HCl buffer solution pH 7.4 to a concentration of ≥9 × 10^7^ cells/mL and the resultant cell pellets were kept in an ice bath and homogenized using a sonication probe. The total protein content in the homogenized crude enzyme was not less than 75 µg protein equivalent in this 5α-reductase inhibitory assay, which was measured using the Pierce bicinchoninic acid (BCA) protein assay (Pierce, Rockford, IL, USA).

#### 3.4.2. Enzymatic 5α-Reductase Inhibition Assay

The in vitro inhibitory activity of the samples against the conversion of testosterone to DHT by 5α-reductase was carried out according to the method described by Srivilai et al. [55]. The DHT formation after the enzymatic reaction was determined using liquid chromatography mass spectrometry (LC-MS) to measure 5α-reductase activity. Curcumin and finasteride, which have been reported as 5α-reductase inhibitors [43,56], were used as the positive controls. In the assay, the reaction was performed in U-shaped 96-deep-well plates covered by well-cap mats to create a solution that contained 10 µL of the tested sample dissolved in dimethyl sulfoxide (DMSO), 20 µL of testosterone (34.7 µM in propylene glycol and water), and 50 µL of *β*-nicotinamide adenine dinucleotide phosphate (NADPH, 1 mM in tris-HCl buffer pH 7.4). The enzymatic reaction was started by adding 80 µL of homogenized crude enzyme (equivalent to 75 µg protein), and the final volume was adjusted to 200 µL by adding 40 µL of tris-HCl buffer pH 7.4. The reaction was maintained in a water bath with a shaker at 37 °C for 60 min and then the reaction was stopped by adding 300 µL of hydroxylamine hydrochloride (HM) (10 mg/mL in 80% *v*/*v* ethanol). The solution was then incubated for another 60 min at 60 °C to completely derivatize all the DHT that was produced. After incubation, the solution was centrifuged at 1700× *g* for 10 min and the supernatant was collected for quantitated DHT production by LC-MS. Two control groups, C_0_ and C_60_, were prepared with all the solutions, including 10 µL of DMSO, but no test sample. Control group C_0_ was stopped (by adding HM) before the enzymatic incubation at 0 min, while the control group C_60_ continued with the enzymatic reaction and was stopped after 60 min of incubation. The DHT production was determined using LC-MS. The inhibition of tested sample was determined by measuring the area under the curve (AUC) of extracted ion chromatogram (EIC) of derivatized-DHT (*m*/*z* [M + H]^+^, 306.2428) which was used to calculate the enzymatic reaction inhibition by the following Equation (1):

(1) $$5\alpha -\mathrm{reductase}\;\mathrm{inhibitory}\;\mathrm{activity}\;\left(\%\right)=\;\left[1\;-\frac{\mathrm{AUC}\;\mathrm{of}\;\mathrm{sample}-\mathrm{AUC}\;\mathrm{of}\;C_{0}}{\mathrm{AUC}\;\mathrm{of}\;C_{60}\;-\mathrm{AUC}\;\mathrm{of}\;C_{0}}\right]\;\times 100$$

### 3.5. Quantitative HPLC Analysis of 5α-Reductase Inhibitors from T. grandis Leaf Extract

#### 3.5.1. Reference Solutions

Compounds **1** and **2**, the two isolated 5α-reductase inhibitors (purity 99%), were accurately weighed and dissolved in methanol. Further dilution was carried out using methanol as the diluting solvent to achieve the desired concentration. All standard solutions were filtered through a 0.45 µm nylon membrane before being injected into the HPLC system.

#### 3.5.2. Chromatographic Conditions

The HPLC apparatus was an Agilent 1260 infinity equipped with a G1315D HPLC diode array detector (Agilent Technologies, Santa Clara, CA, USA). The chromatographic separation was performed on a reversed-phase Phenomenex Luna C18(2) column (150 mm × 4.6 mm, 5 µm particle size). A mixture solution of acetonitrile and 0.1% (*v*/*v*) formic acid in purified water (85:15 *v*/*v*) was used as the mobile phase. The isocratic elution system was programmed with a 0.8 mL/min flow rate at room temperature and the UV chromatogram was recorded at 220 nm. The injection volume was 20 µL.

#### 3.5.3. Validation of HPLC Method

According to the International Council for Harmonisation of Technical Requirements for Registration of Pharmaceuticals for Human Use (ICH) guidelines [57], the HPLC method that we developed was validated, including the linearity, limit of detection (LOD), the limit of quantification (LOQ), accuracy, and intra-day and inter-day precision. The linearity range of **1** and **2** were determined on eight concentration levels (1.56–200 µg/mL) with triplicate experiments. A calibration curve was created by plotting the mean of the peak areas versus their concentrations and expressed by calculating the slope, y-intercept, and the squared regression coefficient (r^2^). LOD and LOQ under the present chromatographic conditions were calculated using the spiked sample blank method applying the lowest known concentration of standard solutions, calculated in 10 replicates. The LOD and LOQ were determined by calculating the standard deviation of the response (which are usually represented as the concentration of analytes in the sample) based on the signal to noise ratio (S/N) equal 3 for LOD and 10 for LOQ. The accuracy of the method was determined using the spiked sample method. Three different concentration levels of **1** and **2** mixtures (15, 75, and 135 µg/mL) were added to the crude ethanolic extract solution covering the specified range in 25, 50, and 75% of their calibration curve. These experiments were completed in triplicate. The accuracy is expressed as a percentage of recovery, which was calculated from 100 × [(C_spiked_ − C_non-spiked_)/C_standard_], where C is concentration in µg/mL unit.

The precision of the method was verified by repeatability (intra-day precision) and intermediate precision (inter-day precision) studies. These studies were performed by analyzing three concentration levels (20, 75, and 150 µg/mL). Intra-day precision was determined by three replicated analyses of each concentration within 1 day (*n* = 3). Inter-day precision was determined in triplicate for consecutive three days. Precision was expressed as a percentage of relative standard deviation (%RSD) calculated from the (standard deviation/mean × 100). For quantification of the two markers, **1** and **2** in *T. grandis* extracts were calculated from the corresponding calibration curve.

### 3.6. Statistical Analysis

Data were expressed as the means ± standard deviation (SD) of at least triplicate experiments. Statistical comparisons were analyzed using one-way analysis of variance (ANOVA), followed by Duncan’s test. *p* < 0.05 was considered statistically significant.

## 4. Conclusions

This study has shown, for the first time, the isolation and identification of 5α-reductase inhibitors; two eperuane-type diterpenes (**1** and **2**) and a lupane-type triterpene (**3**) from the leaf extract of *T. grandis*. The HPLC analysis of the two potent 5α-reductase inhibitors, **1** and **2**, were developed, validated, and successfully applied for these compounds in *T. grandis* leaf extracts. The extraction of *T. grandis* leaves with 95% ethanol resulted in a higher volume of extract yielded which contained higher concentrations of **1** and **2**, as well as exhibiting greater 5α-reductase inhibitory activity, than the hexane extract. Our discovery suggests that **1** and **2** can be bioactive markers for the further development of *T. grandis* leaf extract as an ingredient in the cosmeceutical products for hair loss treatment.

## Figures and Tables

**Table 1:** IC_50_ values against 5α-reductase of the three isolated compounds (**1–3**) and two 5α-reductase inhibitors. The data are expressed as the means ± standard deviation (SD) of triplicate experiments.

Samples	IC_50_ (µg/mL)	IC_50_ (µM)
Isolated Compounds		
(+)-Eperua-8,13-dien-15-oic acid (1)	4.31 ± 0.87	14.19 ± 2.87 ^a^
(+)-Eperua-7,13-dien-15-oic acid (2)	4.45 ± 0.10	14.65 ± 0.31^a^
Lupeol (3)	>170	>400
Positive controls [43]		
Curcumin	4.95 ± 0.15	13.40 ± 0.40 ^a^
Finasteride	0.28 ± 0.01	0.73 ± 0.03 ^b^

The mean values of IC_50_ (µM) from each sample were compared using one-way ANOVA followed by Duncan’s test. ^a, b^ Values not sharing the same letter are significantly different from another (*p* < 0.05).

**Table 2:** Method validation parameters for the determination of **1** and **2** by the proposed HPLC method.

Parameters	Values	
	1	2
Linearity range	1.56–200 µg/mL	1.56–200 µg/mL
Regression equation	y = 63.483x + 40.465	y = 75.954x + 20.8
Correlation coefficient (r^2^)	0.9997	0.9995
Limits of detection (LOD)	0.09 µg/mL	0.06 µg/mL
Limits of quantification (LOQ)	0.30 µg/mL	0.20 µg/mL

**Table 3:** Accuracy (% recovery) and intra- and inter-day precisions of **1** and **2** by the proposed HPLC method.

Accuracy			Precision				
Concentration (µg/mL)	Recovery (%) ± SD		Concentration (µg/mL)	RSD (%)			
				Intra-Day ^a^		Inter-Day ^b^	
	1	2		1	2	1	2
15	92.78 ± 0.77	99.34 ± 3.06	20	0.46	2.33	1.90	2.10
75	100.61 ± 0.86	98.20 ± 0.77	75	0.11	0.50	1.02	1.51
135	97.13 ± 2.71	98.94 ± 1.26	150	0.02	0.60	0.13	1.15

^a^ Intra-day at three times in one day. ^b^ Inter-day on three different days.

**Table 4:** The contents of two 5α-reductase inhibitors (**1** and **2**) in hexane and ethanolic extracts of *T. grandis* leaf and IC_50_ values against 5α-reductase of *T. grandis* leaf extracts. The data are expressed as the means ± standard deviation (SD) of triplicate experiments.

Samples	Contents (% *w*/*w*)		IC_50_ against 5α-Reductase (µg/mL)
	1	2	
Hexane extract	5.60 ± 0.05	3.23 ± 0.03	26.45 ± 0.69
Ethanolic extract	6.18 ± 0.12 ^a^	3.83 ± 0.04 ^a^	23.91 ± 0.17 ^a^

^a^ *p* < 0.05, significantly different compared with the hexane extract.

## Data Availability

The data presented in this study are available on request from the corresponding author.

## Supplementary Materials

The following supporting information can be downloaded at: https://www.mdpi.com/article/10.3390/molecules27092893/s1, Figure S1: ^1^H-NMR spectrum of **1** (400 MHz, CDCl_3_), Figure S2: ^13^C-NMR spectrum of **1** (100 MHz, CDCl_3_), Figure S3: HMBC spectrum of **1** (400 MHz for ^1^H and 100 MHz, for ^13^C, CDCl_3_), Figure S4: FT-IR (ATR mode) spectrum of **1**, Figure S5: HRESI-MS (negative ion mode) spectrum of **1**, Figure S6: ^1^H-NMR spectrum of **2** (400 MHz, CDCl_3_), Figure S7: ^13^C-NMR spectrum of **2** (100 MHz, CDCl_3_), Figure S8: HMBC spectrum of **2** (400 MHz for ^1^H and 100 MHz, for ^13^C, CDCl_3_), Figure S9: FT-IR (ATR mode) spectrum of **2**, Figure S10: HRESI-MS (negative ion mode) spectrum of **2**, Figure S11: ^1^H-NMR spectrum of **3** (400 MHz, CDCl_3_), Figure S12: ^13^C-NMR spectrum of **3** (100 MHz, CDCl_3_), Figure S13: FT-IR (ATR mode) spectrum of **3** and an expanded region of the spectrum (a) chemical shift at 35.0–60.0 ppm and (b) chemical shift at 12.0–32.0 ppm, Figure S14: EI-MS spectrum of **3**, Table S1: NMR data of **1** and **2** (in CDCl_3_) measured at 100 (^13^C) and 400 (^1^H) MHz, Table S2: NMR data of **3** (in CDCl_3_) measured at 100 (^13^C) and 400 (^1^H) MHz.

## Sample Availability

Samples of the compounds **1**–**3** are available from the authors.
