# Supplementary material for: Isolation and HPLC Quantitative Determination of 5α-Reductase Inhibitors from Tectona grandis L.f. Leaf Extract
Source: Molecules. 2022 Apr 30;27(9):2893. doi: 10.3390/molecules27092893 (PMC9101728; doi:10.3390/molecules27092893)
Supplement: Supplementary file 1 [file molecules-27-02893-s001.zip › molecules-1669800_Supplementary.pdf]

molecules

Supplementary Files

Isolation and HPLC quantitative determination of 5α-reductase inhibitors from *Tectona grandis* L.f. leaf extract

Kamonlak Insumrong [1], Kornkanok Ingkaninan [2], Neti Waranuch [3], Nutchaninad Tanuphol [2], Wudtichai Wisuitiprot [4], Trinop Promgool [3], and Nungruthai Suphrom [1,5,*]

1 Department of Chemistry, Faculty of Science, Naresuan University, Phitsanulok 65000, Thailand; kamonlaki59@nu.ac.th
2 Centre of Excellence in Cannabis Research, Department of Pharmaceutical Chemistry and Pharmacognosy, Faculty of Pharmaceutical Sciences and Center of Excellence for Innovation in Chemistry, Naresuan University, Phitsanulok 65000, Thailand; k_ingkaninan@yahoo.com (K.I.); nutchaninadt62@nu.ac.th (N.T.)
3 Cosmetics and Natural Products Research Center and Center of Excellence for Innovation in Chemistry, Department of Pharmaceutical Technology, Faculty of Pharmaceutical Sciences, Naresuan University, Phitsanulok 65000, Thailand; netiw@nu.ac.th (N.W.); tpromgool@gmail.com (T.P.)
4 Department of Thai Traditional Medicine, Sirindhorn College of Public Health, Phitsanulok 65130, Thailand; wisuitiprot@hotmail.com
5 Department of Chemistry, Faculty of Science and Center of Excellence for Innovation in Chemistry, Naresuan University, Phitsanulok 65000, Thailand; suphrom.n1@gmail.com
* Correspondence: suphrom.n1@gmail.com; Tel.: +66-92-257-31-45; Fax: +66-55-96-34-01

Supplementary Materials

Figure S1: ^{1}H–NMR spectrum of **1** (400 MHz, $CDCl_3$)

Figure S2: ^{13}C–NMR spectrum of **1** (100 MHz, $CDCl_3$)

Figure S3: HMBC spectrum of **1** (400 MHz for ^{1}H and 100 MHz, for ^{13}C, $CDCl_3$)

Figure S4: FT–IR (ATR mode) spectrum of **1**

Figure S5: HRESI–MS (negative ion mode) spectrum of **1**

Figure S6: ^{1}H–NMR spectrum of **2** (400 MHz, $CDCl_3$)

Figure S7: ^{13}C–NMR spectrum of **2** (100 MHz, $CDCl_3$)

Figure S8: HMBC spectrum of **2** (400 MHz for ^{1}H and 100 MHz, for ^{13}C, $CDCl_3$)

Figure S9: FT–IR (ATR mode) spectrum of **2**

Figure S10: HRESI–MS (negative ion mode) spectrum of **2**

Figure S11: ^{1}H–NMR spectrum of **3** (400 MHz, $CDCl_3$)

Figure S12: ^{13}C–NMR spectrum of **3** (100 MHz, $CDCl_3$)

Figure S13: FT–IR (ATR mode) spectrum of **3** and an expanded region of the spectrum (a) chemical shift at 35.0-60.0 ppm and (b) chemical shift at 12.0-32.0 ppm

Figure S14: EI–MS spectrum of **3**

Table S1: NMR data of **1** and **2** (in $CDCl_3$) measured at 100 (^{13}C) and 400 (^{1}H) MHz

Table S2: NMR data of **3** (in $CDCl_3$) measured at 100 (^{13}C) and 400 (^{1}H) MHz

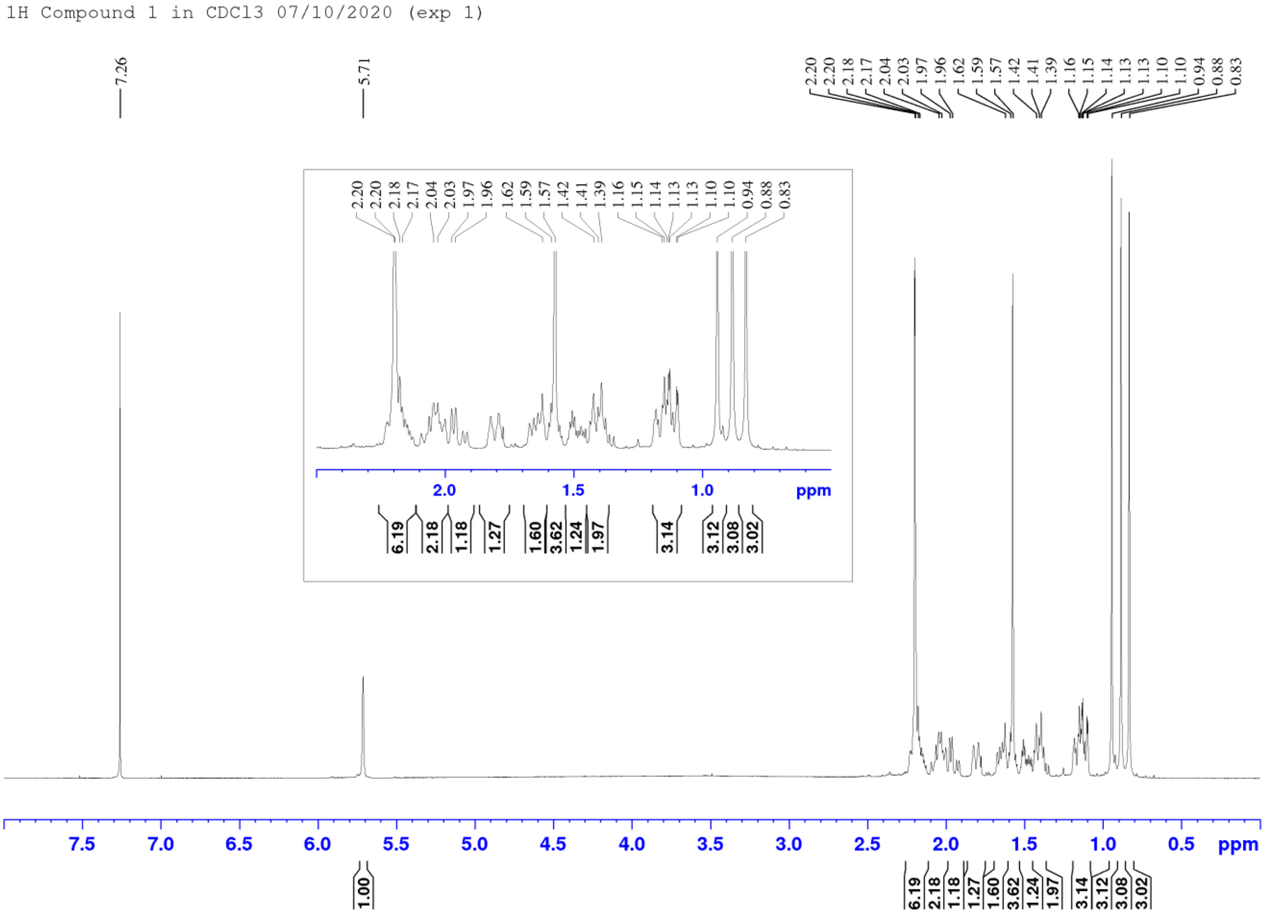

Figure S1. ^{1}H–NMR spectrum of **1** (400 MHz, $CDCl_3$)

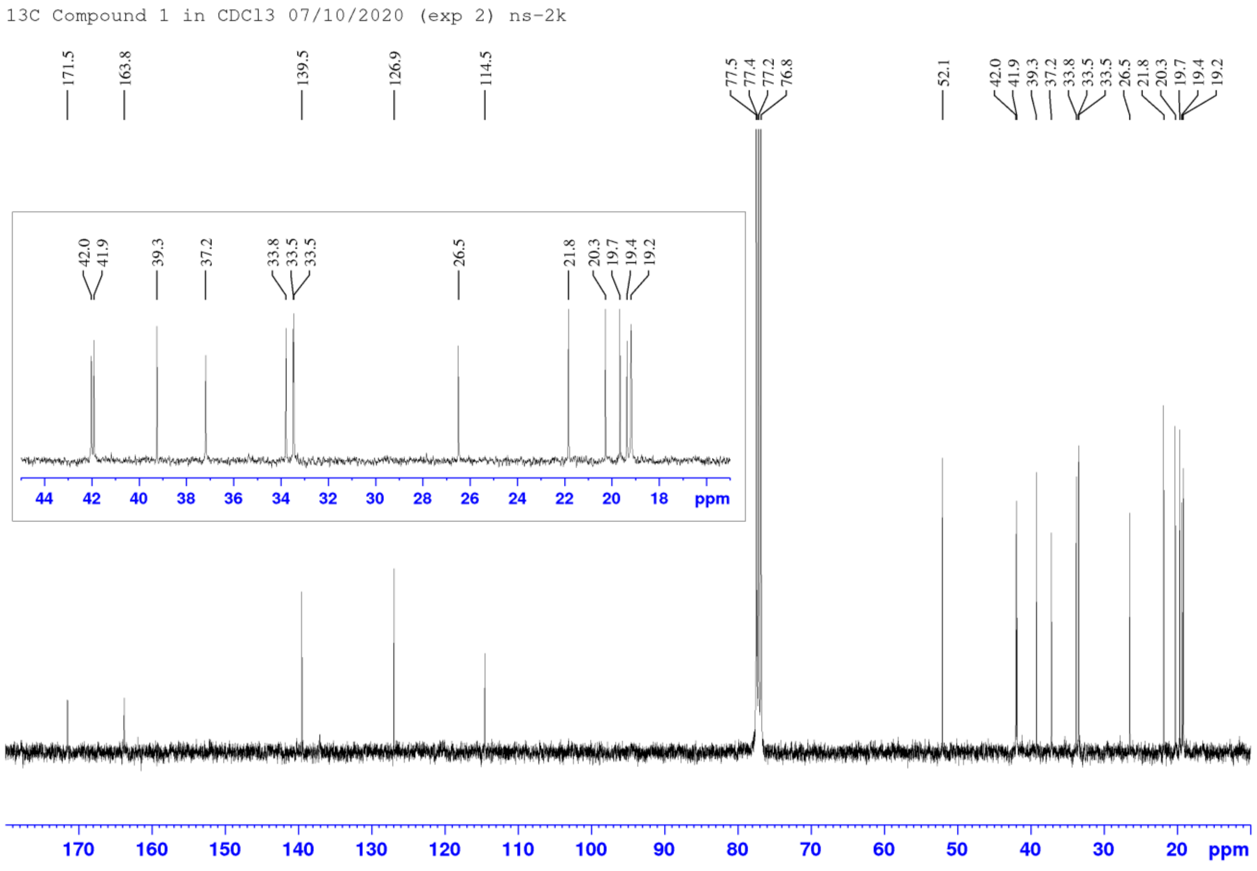

Figure S2. ^{13}C–NMR spectrum of **1** (100 MHz, $CDCl_3$)

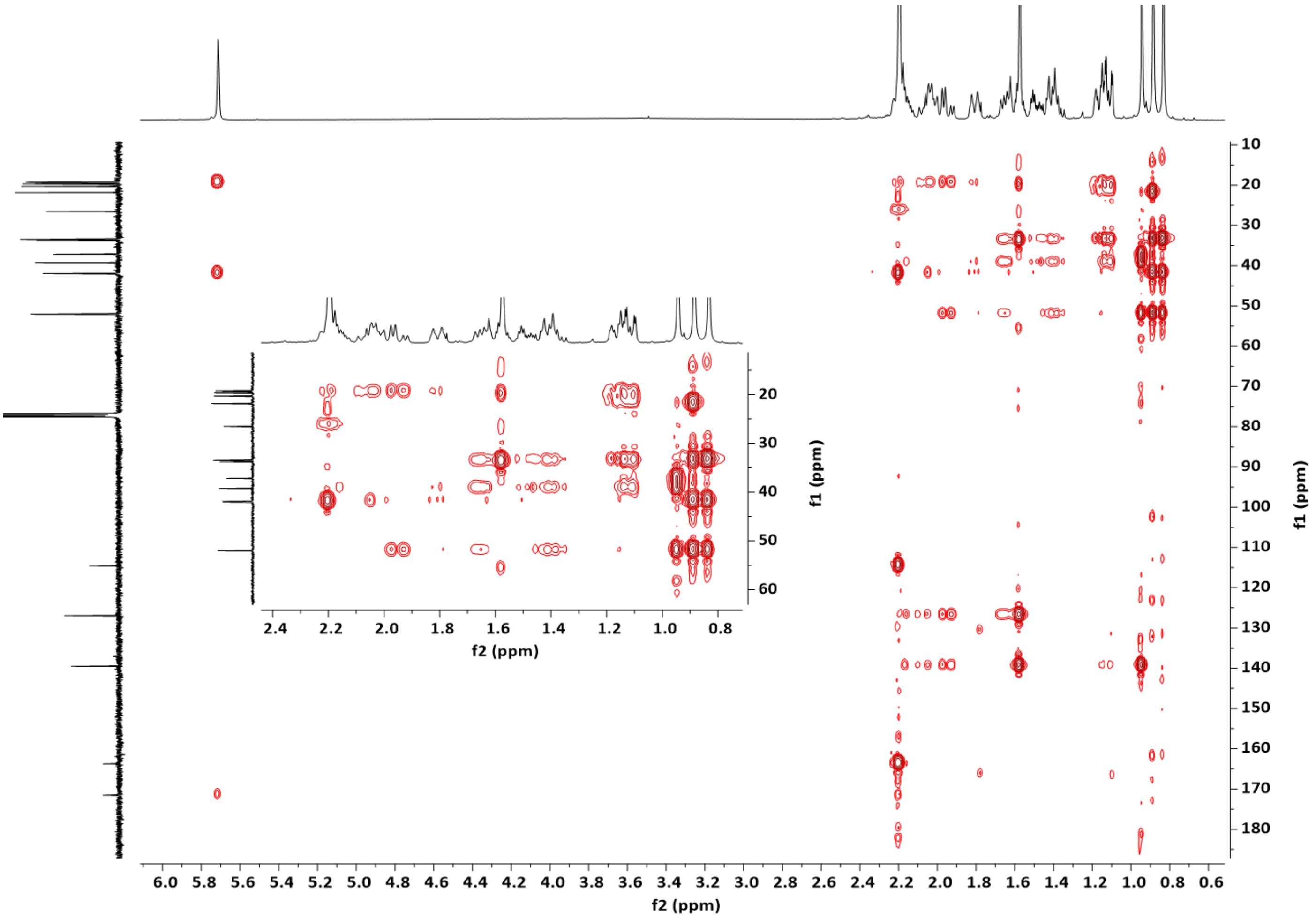

Figure S3: HMBC spectrum of **1** (400 MHz for ^{1}H and 100 MHz, for ^{13}C, $CDCl_3$)

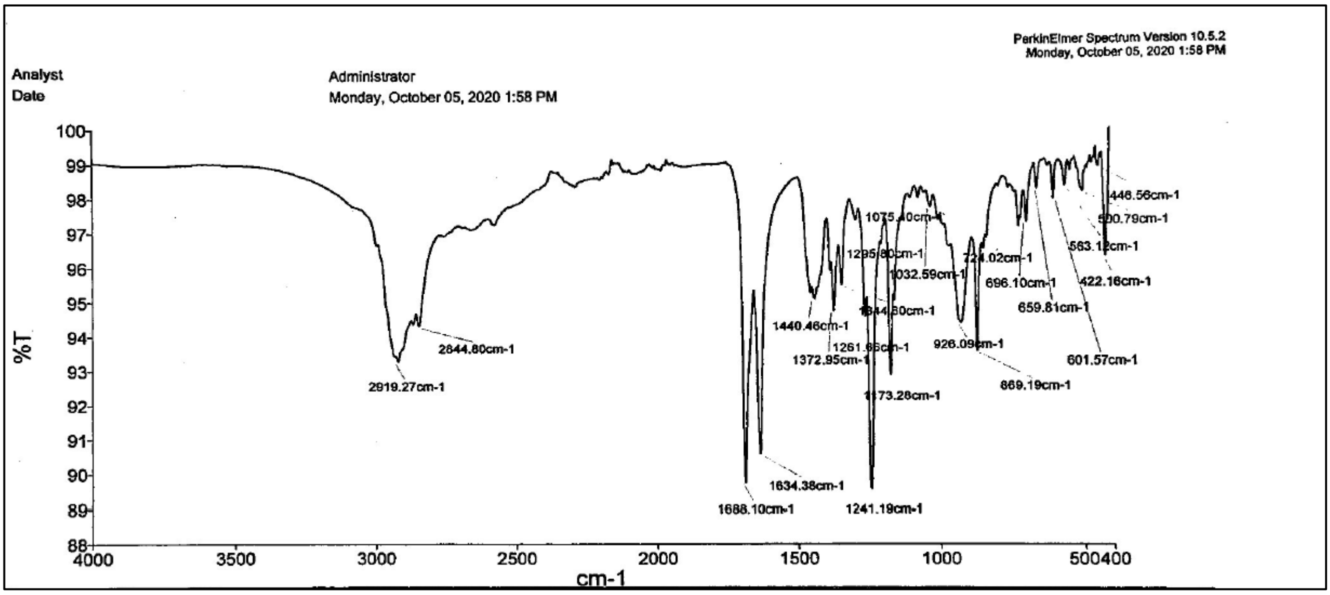

Figure S4: FT–IR (ATR mode) spectrum of **1**

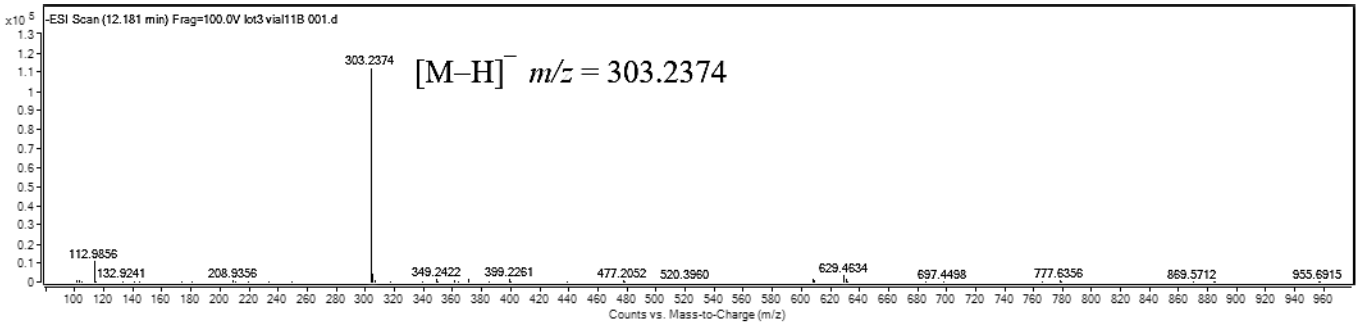

Figure S5. HRESI–MS (negative ion mode) spectrum of **1**

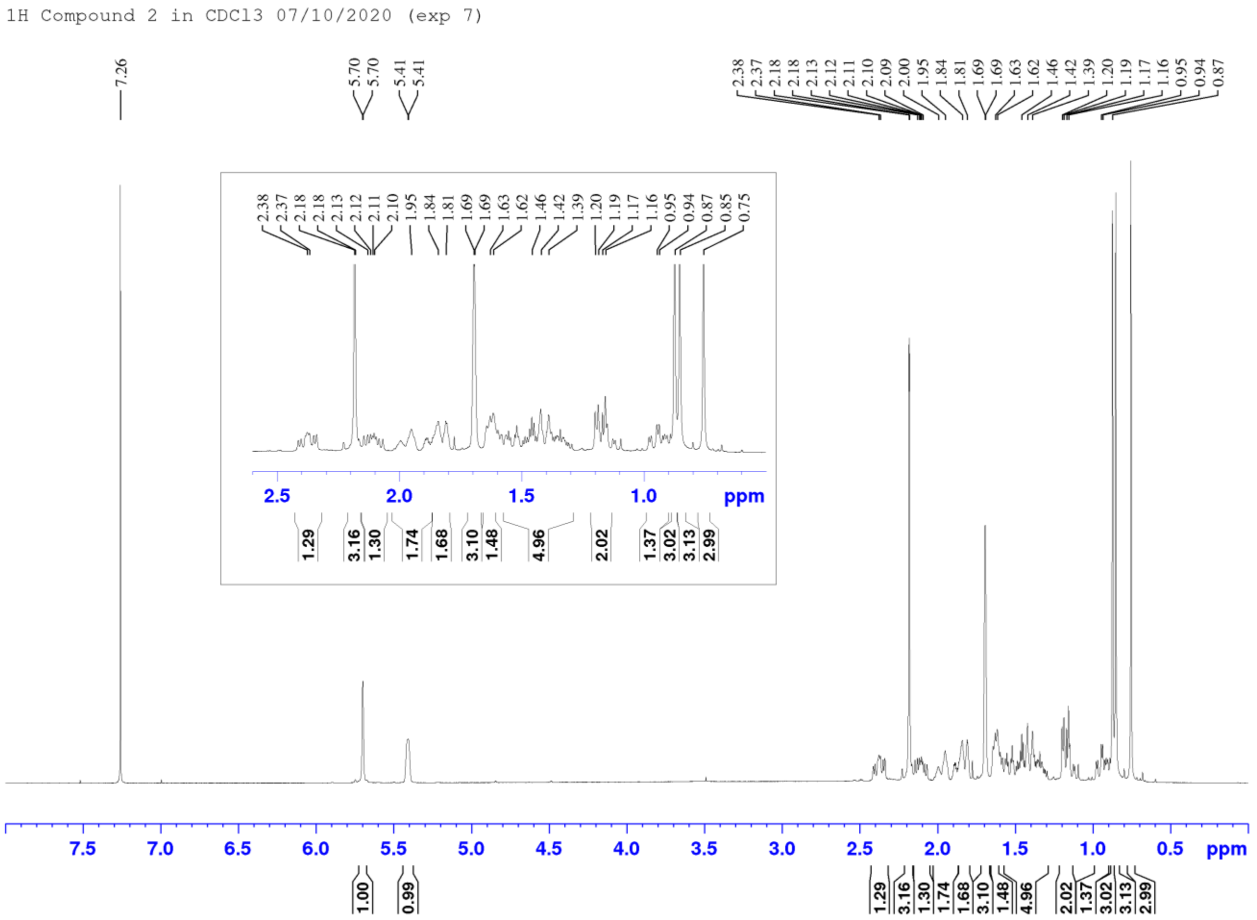

Figure S6. ^{1}H–NMR spectrum of **2** (400 MHz, $CDCl_3$)

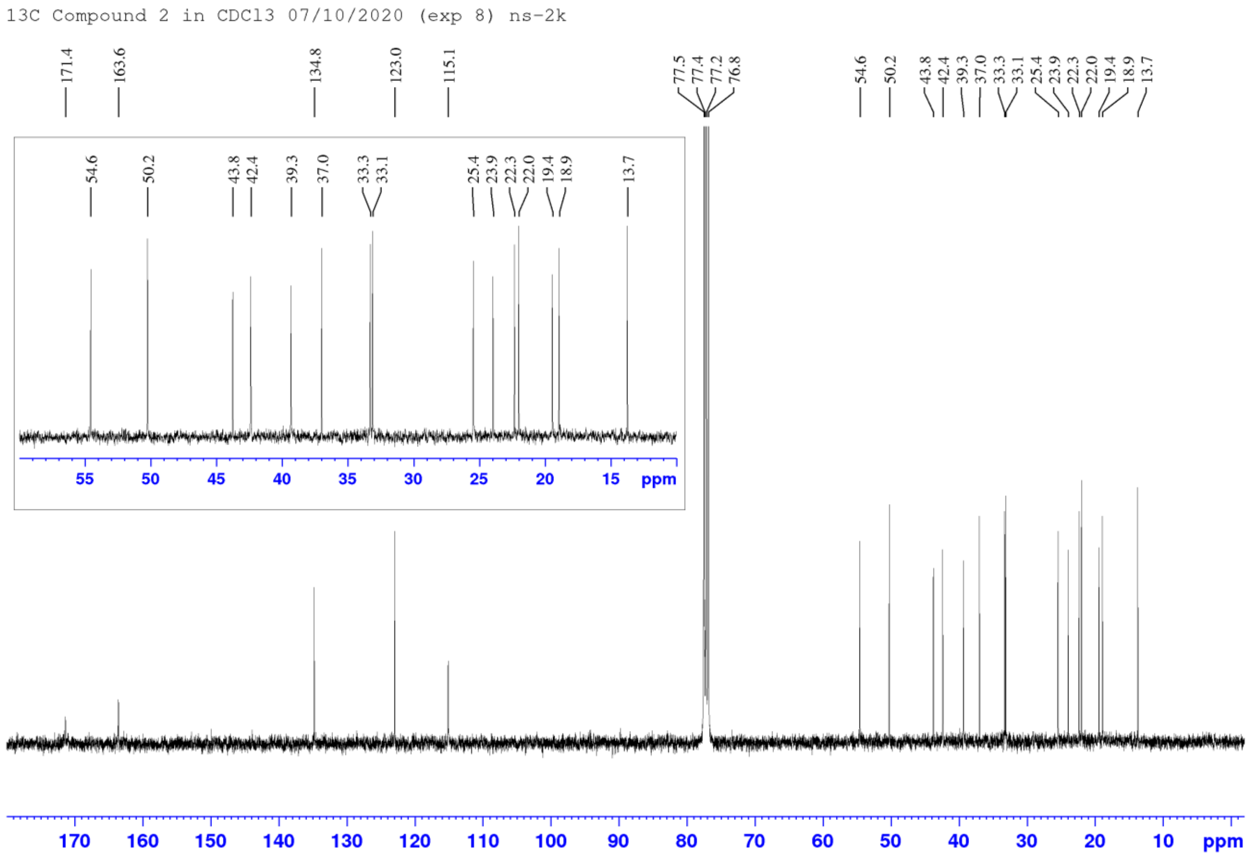

Figure S7. ^{13}C–NMR spectrum of **2** (100 MHz, $CDCl_3$)

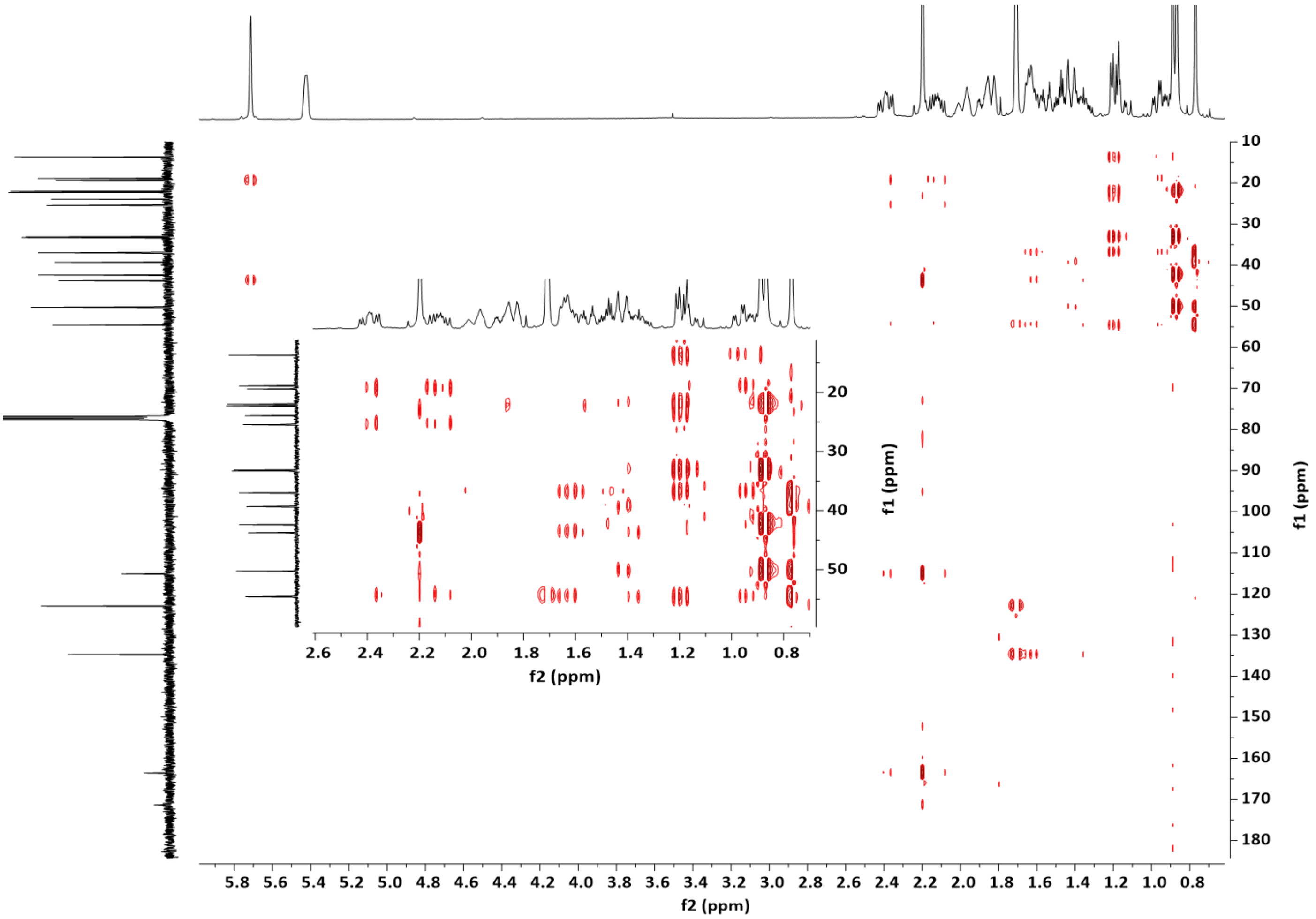

Figure S8: HMBC spectrum of **2** (400 MHz for ^{1}H and 100 MHz, for ^{13}C, $CDCl_3$)

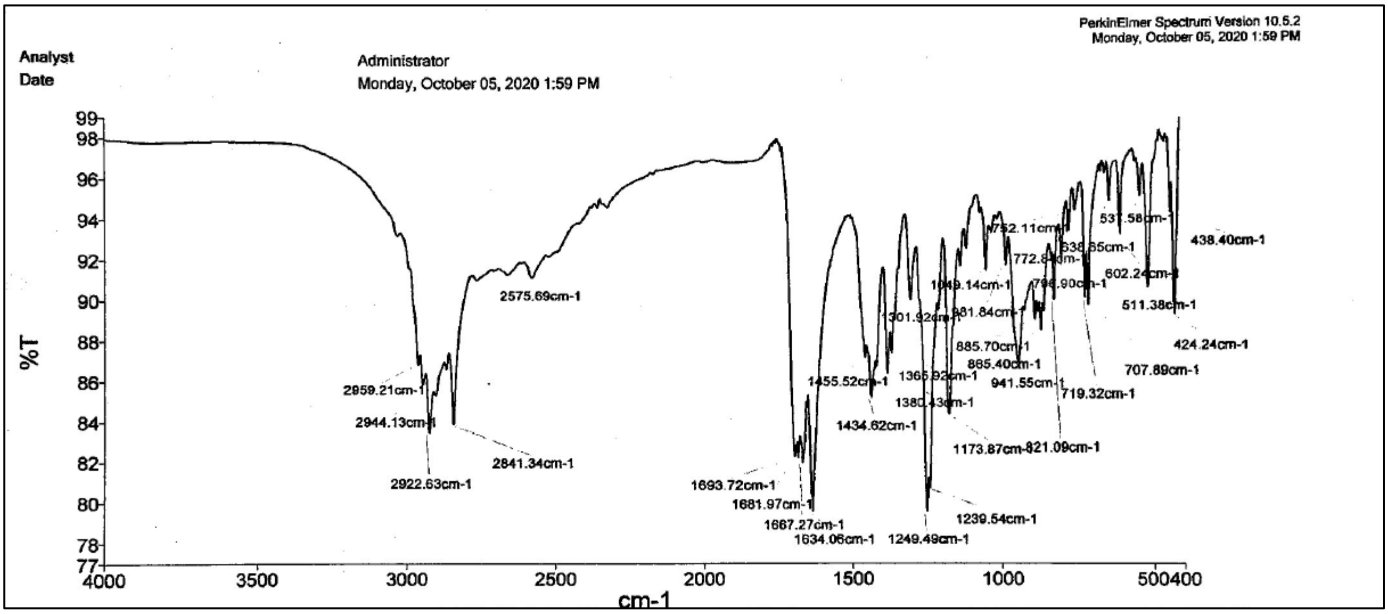

Figure S9: FT–IR (ATR mode) spectrum of **2**

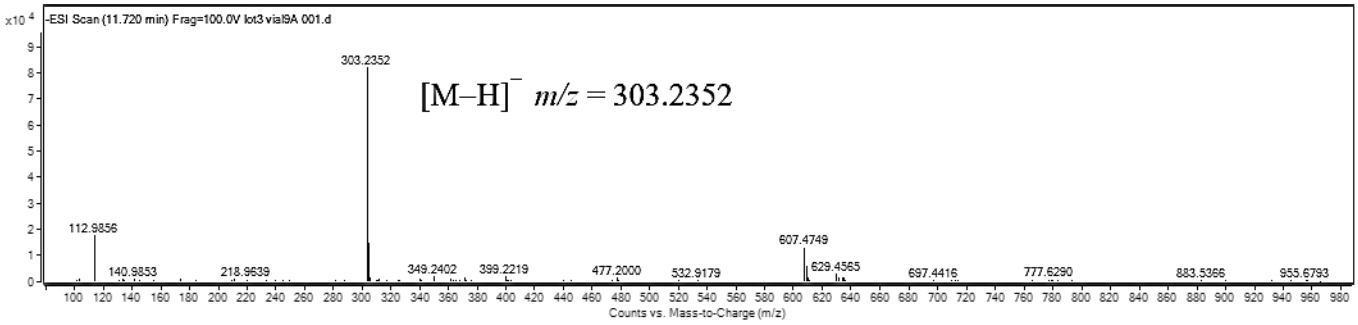

Figure S10: HRESI–MS (negative ion mode) spectrum of **2**

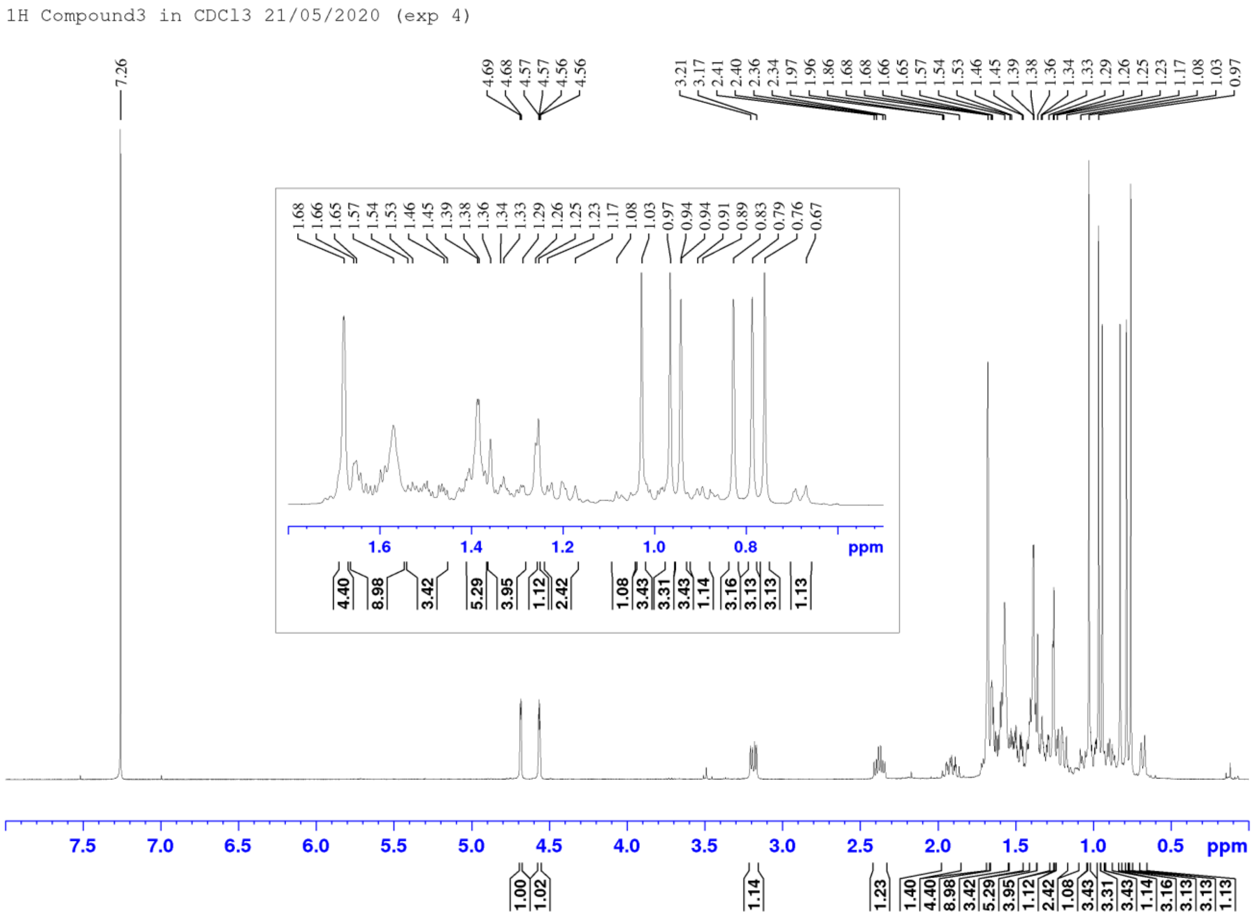

Figure S11. ^{1}H–NMR spectrum of **3** (400 MHz, $CDCl_3$)

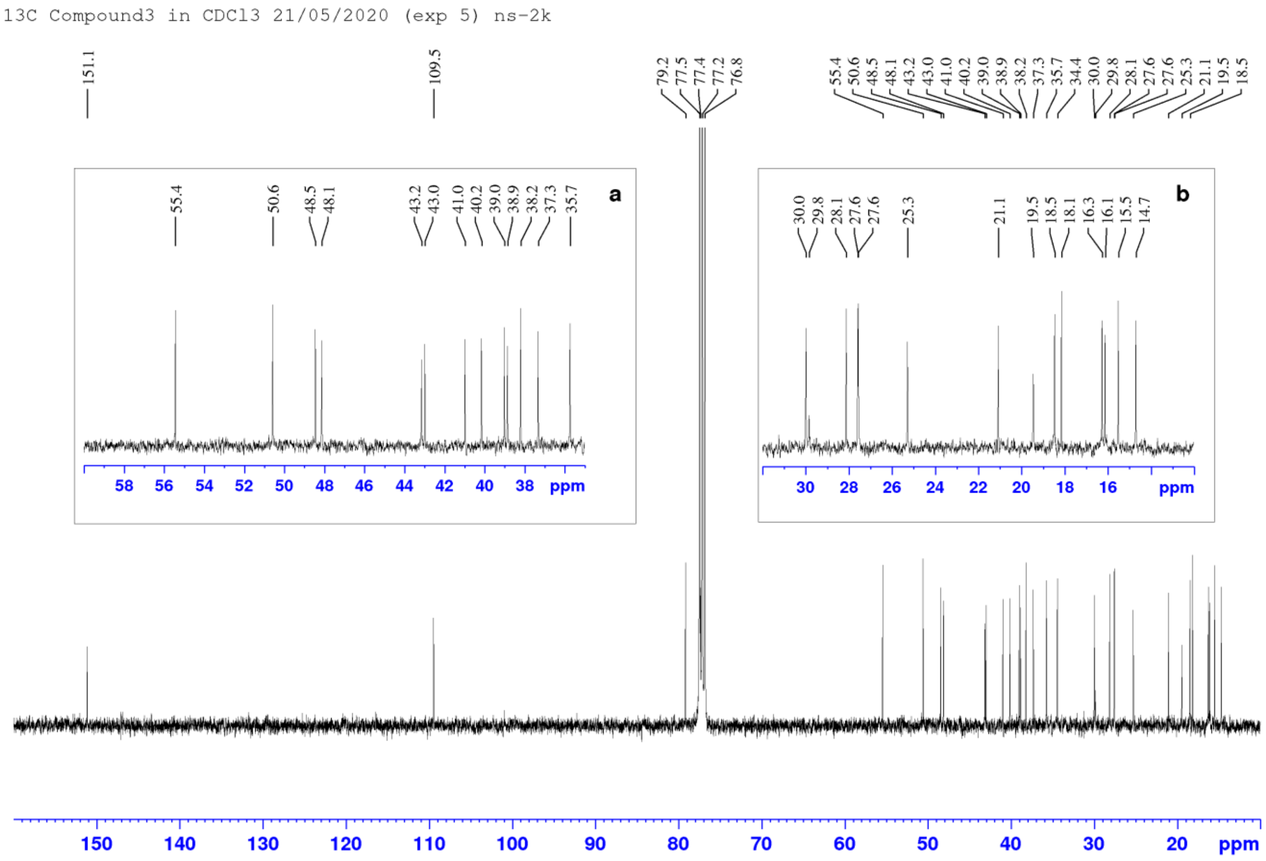

Figure S12. ^{13}C–NMR spectrum of **3** (100 MHz, $CDCl_3$) and an expanded region of the spectrum (a) chemical shift at 35.0-60.0 ppm and (b) chemical shift at 12.0-32.0 ppm

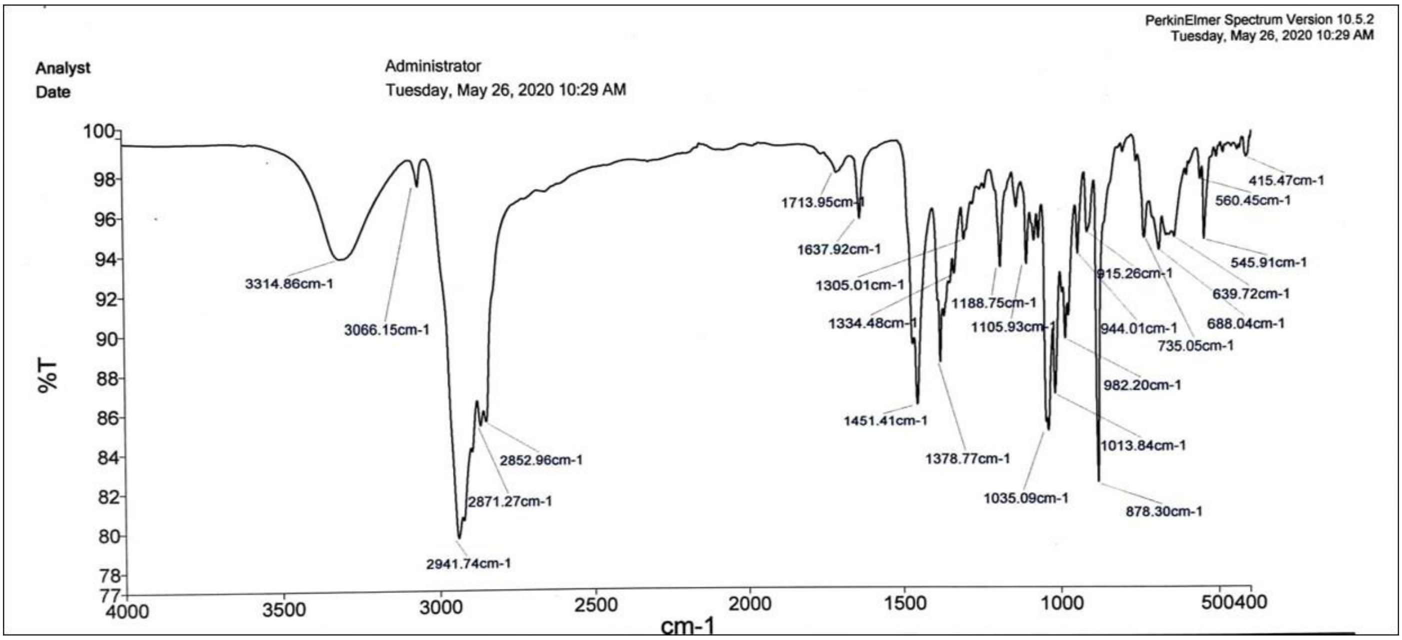

Figure S13: FT–IR (ATR mode) spectrum of **3**

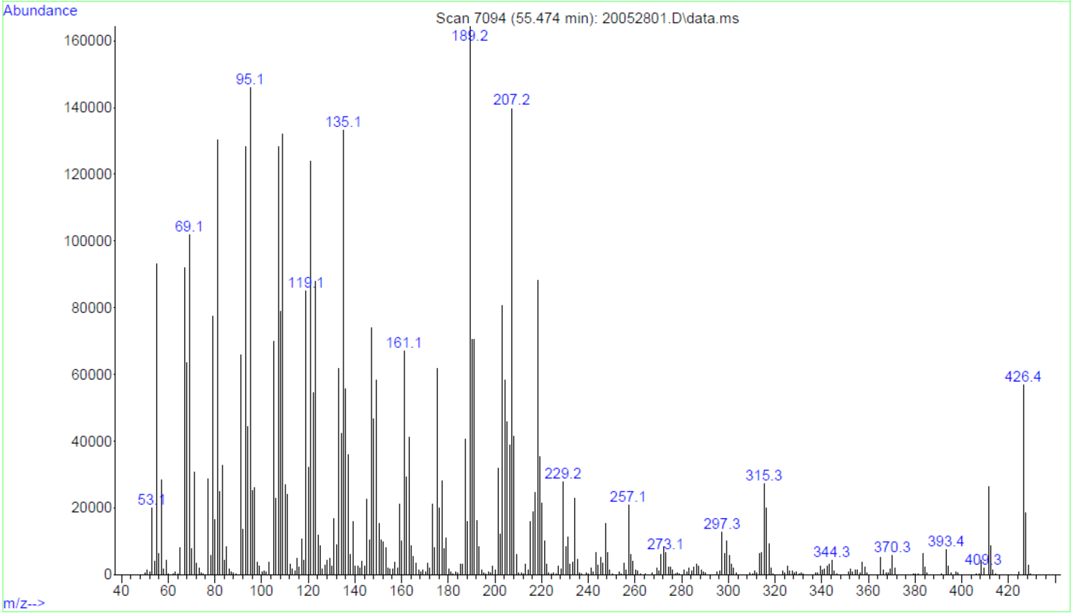

Figure S14: EI–MS spectrum of **3**

Table S1 NMR data of **1** and **2** (in $CDCl_3$) measured at 100 (^{13}C) and 400 (^{1}H) MHz

Position	**1**		**2**	
	^{13}C (ppm)[a], mult.[b]	^{1}H (ppm)[a], mult. (*J* in Hz)	^{13}C (ppm)[a], mult.[b]	^{1}H (ppm)[a], mult. (*J* in Hz)
1	37.2, CH_2	1.14, *m*	39.3, CH_2	0.94, *m*
		1.81, *m*		1.83, *m*
2	19.2, CH_2	1.48, *m*	18.9, CH_2	1.42, *m*
3	41.9, CH_2	1.14, *m*	42.4, CH_2	1.17, *m*
		1.40, *m*		1.42, *m*
4	33.5, C	–	33.1, C	–
5	52.1, CH	1.14, *m*	50.2, CH	1.17, *m*
6	19.2, CH_2	1.64, *m*	23.9, CH_2	1.95, *m*
7	33.8, CH_2	1.97, *m*	123.0, CH	5.41, *br s*
		2.03, *m*		
8	126.9, C	–	134.8, C	–
9	139.5, C	–	54.6, CH	1.62, *m*
10	39.3, C	–	37.0, C	–
11	26.5 CH_2	2.04, *m*	25.4, CH_2	1.42, *m*
		2.20, *m*		
12	42.0, CH_2	2.20, *m*	43.8, CH_2	2.12, *m*
				2.37, *m*
13	163.8, C	–	163.6, C	–
14	114.5, CH	5.71, *br s*	115.1, CH	5.70, *br s*
15	171.5, C	–	171.4, C	–
16	19.4, CH_3	2.20, *d* (1.1)	19.4, CH_3	2.18, *d* (1.1)
17	19.7, CH_3	1.57, *s*	22.3, CH_3	1.69, *br s*
18	33.5, CH_3	0.88, *s*	33.3, CH_3	0.87, *s*
19	21.8, CH_3	0.83, *s*	22.0, CH_3	0.85, *s*
20	20.3, CH_3	0.94, *s*	13.7, CH_3	0.75, *s*

[a] Assignments were based on ^{1}H, ^{13}C, and HMQC experiments; [b] Multiplicities were established by DEPT experiment.

Table S2 NMR data of **3** (in $CDCl_3$) measured at 100 (^{13}C) and 400 (^{1}H) MHz

Position	^{13}C (ppm)	^{1}H (ppm), mult. (*J* in Hz)
1	38.9	0.89, 1.65, *m*
2	27.6	1.62, *m*
3	79.2	3.18, *dd* (11.2, 5.0)
4	39.0	-
5	55.4	0.67, *m*
6	18.5	1.36, 1.53, *m*
7	34.4	1.39, *m*
8	41.0	-
9	50.6	1.33, *m*
10	37.3	-
11	21.1	1.31, 1.40, *m*
12	25.3	1.08, 1.62, *m*
13	38.2	1.65, *m*
14	43.0	-
15	27.6	1.05, 1.60, m
16	35.7	1.50, *m*
17	43.2	-
18	48.5	1.38, *m*
19	48.1	2.38, *dt* (11.0, 5.8)
20	151.1	-
21	30.0	1.29, *m*
22	40.2	1.17, *m*
23	28.1	0.96, *s*
24	15.5	0.76, *s*
25	16.1	0.83, *s*
26	16.3	1.03, *s*
27	14.7	0.94, *s*
28	18.1	0.79, *s*
29	109.5	4.56, *br d*
		4.69, *br d*
30	19.5	1.68, *s*
